# Concerning Third Molars

**Published:** 1891-01

**Authors:** C. A. Brackett

**Affiliations:** Newport, R. I.


### CONCERNING THIRD MOLARS.[1]

BY DR. C. A. BRACKETT, NEWPORT, R. I.

When our good Executive Committee honored me with an invitation to read a paper before you, I was moved by a number of considerations to take the subject which has been announced. I have little to say in theory, but there are connected with the third molar practical points whose careful consideration may be profitable for us, and through us of great advantage to those who come to us seeking relief from some form of suffering which a third molar may occasion.

The third molar is a tooth against which, justly or unjustly, much is said. There is a popular prejudice against it as "a black sheep in the flock, even shorn, which comes up from the washing." Patients say, "If that is a wisdom tooth, I don't want to have anything done to try to save it, because it is sure not to last. Wisdom teeth are decayed before they are half through the gum; they are forever coming, and all the while are a source of torment. From beginning to end there is nothing good about them, and I don't see why we have wisdom teeth anyway."

It must be confessed that there are numerous instances in which such statements are not a distortion of the truth. The teeth are valueless, and worse than valueless, and often patients do suffer from them seriously and extremely; but to condemn the third molar *in toto* and indiscriminately is not warranted. There is another aspect of the subject, and in fairness it should be presented.

With a view of determining the relative frequency of decay with the different teeth, Dr. Magitot has prepared a table, from the results of his examinations of permanent teeth for caries, showing that, of ten thousand instances, but three hundred and sixty were of the third molar. By Magitot's table the order of frequency of caries of the different teeth is as follows: Lower first molar, upper first molar, lower second molar, upper first bicuspid, upper second bicuspid, upper lateral incisor, upper second molar, upper central incisor, lower second bicuspid, upper canine, lower first bicuspid, upper third molar, lower third molar, lower canine, the two lower incisors.

This showing is remarkably good, and the position in the scale

---

[1] Read at the meeting of the Academy of Dental Science, Boston, March 8.

of the third molar next to the lower canine is a place of great honor. Were the ten thousand instances of caries equally divided among the different classes of the thirty-two teeth, the four third molars would be entitled to an eighth of ten thousand, or twelve hundred and fifty, instead of the three hundred and sixty which were found. We do not know the ages of Magitot's patients. Presumably the cases were consecutive ones for examination in practice, and doubtless included a fair proportion of patients not of an age to have third molars; but allowing this to be the case, a moment's thought will show us that such is real life, and if we are seeking to get at the proportion of cariously infected third molars to cariously infected other teeth, I do not see that there is reason for believing that there is great fallacy in Magitot's figures.

The late Professor T. B. Hitchcock prepared a table of twenty thousand cases of fillings and of extractions for all reasons, chiefly, of course, for caries, the cases of filling and of extraction being all grouped together. Of these twenty thousand cases, nineteen hundred and twenty-four were third molars. A full eighth would be twenty-five hundred, or nearly thirty-three per cent. more than the actual; but of the twenty thousand, the number of first molars was forty-four hundred and ninety-nine. Without going into too much particularity, the order of frequency of affection by Dr. Hitchcock's table is,—upper and lower teeth being grouped together,—the first molar, the second molar, the second bicuspid, the central incisor, the first bicuspid, the lateral incisor, the third molar, the canine. Classing the upper and lower incisors together modifies the place of each in the scale, but does not affect the relative position of the third molar, which has here again honorable showing, so that after making all allowances for circumstances influencing the figures in the table, the deduction seems fair that the third molar is not among teeth the one most prone to caries.

There are reasons why we might look to find it a better tooth than the others, prominent among these reasons being, of course, the circumstance of its development at a time in the age of the individual when the constructional powers are at their best. Given a good heredity, with no mischief-making mixing of family types, with no disproportion between the size of the teeth and the size of the jaws, with proper diet and proper regimen in all particulars throughout the period of development, it is reasonable to expect to find—and practically we do find in cases of this kind—an approach to perfection of organization and arrangement in which the third molar is no exception to the general rule; and there are, as we all know, instances in which the later rather than the earlier circumstances of growth were most favorable, in which the third molar is the best tooth in the mouth. It is also often an excellent tooth in mouths from which other molars have been early lost, so that it has ample room for development. A beautiful specimen of good formation may be found, in a large proportion of cases, by cutting down upon and extracting a third molar before it has erupted at all, and before it has become a pathological centre through impaction, malposition, or difficulty or delay of eruption. Not all third molars are perfect in formation, but a practical study of the subject will show that a much larger proportion of them are formed perfectly than remain perfect. Many of them are more sinned against than sinning. They are the victims of unfavorable circumstances, of bad environment, of unhygienic conditions, of poor ventilation, imperfect drainage, lacking cleanliness, microbe pollution, malaria,—all of these in the immediate, local sense intrinsic in the investments of the particular teeth, or more generally in the system of the possessor of the teeth or in his surroundings in turn, disproportion between the size of the tooth and the place which is provided for it to occupy in the world, slow eruption, overlying gums, surrounding inflammation, and its regular, overhanging cheek, comparative inaccessibility, so that it cannot be reached well by the ordinary means of grace and salvation for teeth,—these and similar elements of the situation should come in for that just estimation which will relieve the tooth itself of much of the odium generally bestowed upon it.

The question whether there is in the frequently-obtaining characteristics of the third molar an illustration and proof of the doctrines of Darwin is an interesting one. I myself believe that we are seeing in the cases of difficult, poor, and dwarfed development of the third molar, and in the not very rare instances of its entire non-appearance among people living under the more artificial conditions, evidences of nature's economy, of the adaptation of means to ends, of the non-development of that which is not demanded. The good development of the third molar in races requiring of their entire masticatory apparatus the most vigorous service is well known.

There is a legend that there have been found fossil remains of an old race of giant men with four well-developed molars in each half of each jaw, but my inquiries thus far have failed to substantiate it. Dr. W. C. Barrett has told me that he has found nothing of the kind. Professor F. W. Putnam, curator of Peabody Museum of American Archæology and Ethnology, has kindly written me this: "In answer to your letter about skulls containing the fourth molar, I can only say that I do not remember ever to have seen such a skull, nor can I recall any mention of one. Dr. Boas, who has examined a great many skulls, is working at our Museum to-day, and he informs me that he has never seen the fourth molar. On the contrary, we both agree that the absence of the third molar is quite a common event."

The not infrequent failure of development of the third molars is a fact to be considered in connection with any suggested situation of the first molars. Doubtless we have all of us seen a number of instances in which, through the sacrifice of the first molars and the non-development of any third molars, the patients have to go through life with but one molar in each quarter of the mouth. Especially is this undesirable state to be guarded against if either parental branch of the child has failed to develop third molars. Such dental peculiarities are very likely to be transmitted from generation to generation.

An old theory which held that carious third molars present in the mouth were a protection to the other teeth through the exhaustion upon themselves of the disintegrating forces appears in the light of modern etiological science rather ridiculous.

The *vis a tergo* of the erupting or erupted third molar may be helpful or harmful to the position of the other teeth according to circumstances. It may serve as a strong buttress to maintain firmly apposed the bulbous proximal surfaces of the other teeth all around the arch; its eruption may close up otherwise existing open spaces; its crowding already overlapping, irregular teeth may markedly increase the deformity of an arch without a key-stone in place; its retarded difficult eruption or efforts at eruption in insufficient space, perhaps itself malposed, is not rarely a source of extreme discomfort, pain, and disability long continued, or recurrent perhaps for years. Irritation, inflammation, and suppuration of its immediate investments are but a portion of the mischief it entails. Happily for escape from lasting disfigurement, abscess about the third molars, whether from crowding or from dead pulp, has less tendency to point externally than have such affections with other lower teeth. Troubles with the throat, with the ear, and other organs of special sense, and many neuralgias in regions that might be supposed to be out of the range of such influence arise in many instances from these third molars.

A practical point in diagnosis to be remembered is that pain often seems to be located more in the peripheral distribution of a given nerve-supply than it really is, farther forward in the mouth, or, through reflex action in situations more or less remote from the actual location of the irritation.

As in all other matters in dentistry, good judgment is needed to determine in a given case whether it is best to conserve or sacrifice a third molar. The probable influence or effect of each procedure is to be studied. The quality of the tooth-structure, tendencies to decay, the relations of the surrounding tissues, the standing of other teeth in the mouth, are all to be regarded. In every procedure involving conservation, sacrifice, or replacement, the antagonism should have careful attention. Without this thoughtfulness there must be in many instances failure to render the best service.

The points which I would especially emphasize are, first, that upon the inception of trouble with a developing third molar, a decision should be made as to whether the tooth is to be retained or not; and second, if the tooth and its situation are such that its room is better than its presence, it should be removed at a very early stage in its history. Disregard of these sound principles and the evasion of the radical operation, where needed, permits patients to suffer much and unnecessarily, and in some few instances subjects them to extreme risk. Cases are on record of even fatal results from pathological developments, having as a nidus an impacted third molar.

If the tooth is to be kept, the application of the principles of depletion, of derivation, of perhaps counter-irritation and cauterization, or gum-section, with or without local or general anæsthesia, is well understood. It cannot be said that the entire list of remedial possibilities is often so speedily and completely successful as we could wish; but in the cases in which their employment is indicated, patience and persistence should ultimately attain the desired end. If the second molar is a bad tooth, while there is a prospect of the third molar becoming a good tooth, in good position, if given an opportunity, the second molar should, of course, be sacrified; but I can hardly conceive a case justifying the loss of a good second molar to relieve the trouble incident to the crowded eruption.

An upper third molar in any position is not often difficult to remove. A lower third molar, if standing nearly erect in the socket, can in the ordinary case of that kind be gotten away with the forceps, even while yet entirely covered with the gum. The difficult cases are mostly of malposed lower teeth, and these are most frequently inclined forward so that the coronal surface presents more or less directly against the distal surface of the second molar. I

have seen a jaw in which a lower third molar was occupying a position directly transverse and horizontal; but any such marked deviation of position is extremely rare.

When once the decision is made that the comfort and well-being of the patient would be favored by the absence of one of these bad third molars, it should be removed with such heroism of operation as the case requires. If the ordinary instruments and procedures for extracting teeth cannot be brought to bear, or will not answer, it should be made such a surgical operation with such appliances as will suffice. The extent and difficulty of these operations are not out of proportion to the seriousness of the troubles to be relieved; and dentists are the ones to operate. The instruments and the details of the operation are to be determined by the peculiarities of each case. Ordinarily, the more difficult of these operations are not to be undertaken without that deliberation and control which are commanded by full etherization. Notwithstanding the patient's inability to open the jaws more than a small fraction of an inch, full opening of the mouth is rarely difficult to accomplish under ether with lever- or screw-power, and a firmly fixed prop seems to retain the opening.

For a third molar, tipped forward and impinging firmly against the distal surface of the second molar, a most helpful next step is the freeing all of that contact by grinding from the third molar with a corundum disk with the engine. This very greatly facilitates the extraction of the third molar, and effectually guards against the deplorable accident—which has occurred—of extracting both second and third molars in the effort to remove the third. The frequent backward curve of the roots of the lower third molar is to be remembered. With the anterior surface free simple lever-power, cautiously applied with a suitably-shaped elevator, may suffice to dislodge the tooth, or to so break up its attachment that the after-removal is easy. Sometimes the process at the sides and overlying the back of the tooth is so thick and strong as to hinder grasping the tooth with a forceps or to prevent its being lifted,—a condition of things particularly embarrassing when the tooth is frail, deeply-decayed, tunnelled, and brittle. In such instances, after suitable dissection of the soft tissues, the bone should be cut away with sharp engine-burs until the difficulty is overcome. Considerable experience has confirmed the conviction that ingenuity and an earnest persistence on the part of the operator, with co-operation on the part of the patient, may surmount the difficulties attendant on the removal of almost any third molar.

However, in these operations well applies the whole of the injunction, "Be bold, be bold, and everywhere be bold; be not too bold." Undue violence, laceration of the soft tissues, and fracture of process, as well as more serious accidents, are all to be guarded against. Any small, loose fragments of process are to be carefully removed, ragged borders are to be ground smooth with corundum stones, and the gum, trimmed with curved scissors if necessary, pressed to overlie in the position most favorable for repair. The patient should understand that one of these operations is very different from an ordinary tooth-extraction, and that the process of recovery, as after any other surgical operation of like extent and involvement of tissue, must take time, and is hardly likely to be free from suffering. Any considerable fracture of process is usually followed by much pain, continuing about a week, and requiring the use of local, sometimes general, anodynes. For the relief of soreness and control of tendency to inflammation, I know of nothing better than very frequent application of the fluid extract of calendula in considerable dilution with water, or fluid extract of hamamelis in full strength. Fetor or tendency to sepsis may in some instances require correction by appropriate means.

Besides such accidents as are possible in tooth-extraction generally, there is in the case of the inferior third molar the peculiar possibility of such injury to the inferior dental nerve as may result in temporary paralysis, or even lasting modification of its functioning. I have seen one such case. It occurred in my own practice in 1874. The very difficult extraction, under ether, of the crooked, hook-like roots of the right inferior third molar for a young lady was followed by loss of sensation in that part of the face. Never having then heard of any such experience, I shared in the patient's anxiety; but, reasoning from the undesired restoration of function after a time following designed simple section of the inferior dental nerve for neuralgia, I felt justified in assuring her that I believed sensation would be restored and that she would be able to note its return in about six weeks. After that interval she did begin to have some prickling sensations comparable to those felt when a limb has "gone to sleep," as the popular experience is; and very slowly some slight further improvement took place till it ceased to be much thought of; but now, after sixteen years, there has not been a return of perfectly normal sensibility. There has never been any motor paralysis or deformity of expression.

Quite an interval elapsed after this operation of mine before I could find that any one else had had a like experience. Mr. S. J. A.

Salter, dental surgeon to Guy's Hospital, in his very practical work, "Dental Pathology and Surgery," published in 1875, gives almost all that I have ever been able to find in print in relation to the subject. On page 354, after expressing his surprise that the text-books of the profession did not make mention of such casualties, he proceeds to describe four cases occurring in his own practice, and he makes allusion to four other cases met by Mr. Bell, and one other by Mr. Holden. The outcome in Mr. Holden's case is not stated. In all of Mr. Bell's cases and in three of Mr. Salter's there was within a short time a return of perfect functioning of the nerve; "and this," he says, "must be considered as the ordinary sequence of the accident." Of Mr. Salter's other case he says, "About six weeks afterwards the patient came to me, and at that time there was scarcely any return of sensation. Six months having elapsed I again saw him; he could then feel when the skin of the lips and chin were touched; but it was not a natural sensation, being a feeling of 'formication,' or what is popularly called 'pins and needles.' From that time to the present he has been often under my hands, and I learn that sensation of the parts has never been completely re-established." That was after an interval of ten years, and the manifestations and history correspond with those in the case of my patient.

The paucity of the literature is evidence that experiences of this kind must be extremely rare,—so rare that the possibility of their occurrence should not debar us from using all reasonable means, with caution of course, to remove impacted lower third molars which are the source of suffering.

[The essayist described several cases of extreme suffering relieved by appropriate treatment of offending third molars, and exhibited a collection of such teeth extracted, showing various peculiarities.]

